# Six decades of lateral flow immunoassay: from determining metabolic markers to diagnosing COVID-19

**DOI:** 10.3934/microbiol.2020018

**Published:** 2020-08-26

**Authors:** Boris G. Andryukov

**Affiliations:** 1 Somov Research Institute of Epidemiology and Microbiology, Vladivostok, Russian Federation; 2 Far Eastern Federal University (FEFU), Vladivostok, Russian Federation

**Keywords:** lateral flow immunoassays (LFIA), clinical laboratory diagnostics, ‘point-of-care testing’, concept

## Abstract

Technologies based on lateral flow immunoassay (LFIA), known in some countries of the world as immunochromatographic tests, have been successfully used for the last six decades in diagnostics of many diseases and conditions as they allow rapid detection of molecular ligands in biosubstrates. The popularity of these diagnostic platforms is constantly increasing in healthcare facilities, particularly those facing limited budgets and time, as well as in household use for individual health monitoring. The advantages of these low-cost devices over modern laboratory-based analyzers come from their availability, opportunity of rapid detection, and ease of use. The attractiveness of these portable diagnostic tools is associated primarily with their high analytical sensitivity and specificity, as well as with the easy visual readout of results. These qualities explain the growing popularity of LFIA in developing countries, when applied at small hospitals, in emergency situations where screening and monitoring health condition is crucially important, and as well as for self-testing of patients. These tools have passed the test of time, and now LFIA test systems are fully consistent with the world's modern concept of ‘point-of-care testing’, finding a wide range of applications not only in human medicine, but also in ecology, veterinary medicine, and agriculture. The extensive opportunities provided by LFIA contribute to the continuous development and improvement of this technology and to the creation of new-generation formats. This review will highlight the modern principles of design of the most widely used formats of test-systems for clinical laboratory diagnostics, summarize the main advantages and disadvantages of the method, as well as the current achievements and prospects of the LFIA technology. The latest innovations are aimed at improving the analytical performance of LFIA platforms for the diagnosis of bacterial and viral infections, including COVID-19.

## 1. Introduction

Successful operation of any clinical laboratory of the world today is unlikely to be possible without test systems based on the lateral flow immunoassay (LFIA) method, which have been used in clinical diagnostics for already 60 years. In some countries of the world, these diagnostic platforms are known as immunochromatographic tests. They are currently a relevant and promising alternative to the existing analytical instrument technologies. These devices are considered as simplified formats of modern biosensors, in which the recognition element is located on the surface of a porous membrane and result is visualized within a few minutes [1]–[4].

These rapid, inexpensive, reliable, and easy-to-use diagnostic platforms have proven their high efficiency in case of limited resources and lack of specially trained personnel. Currently, LFIA tests represent the most promising and dynamically developing segment of the market of rapid *in vitro* diagnostic tools, with an annual cumulative growth in global production of 7.7% [1],[2],[4],[5].

The growing popularity of these test systems for medical care or diagnostics in developing countries, medical institutions, emergency situations, as well as for individual use by patients monitoring their health at home are the major factors that contribute to the continuous development and improvement of this method and to the invention of new-generation formats [2],[6]–[8].

## 2. Lateral flow immunoassay (LFIA)

**Figure 1.**
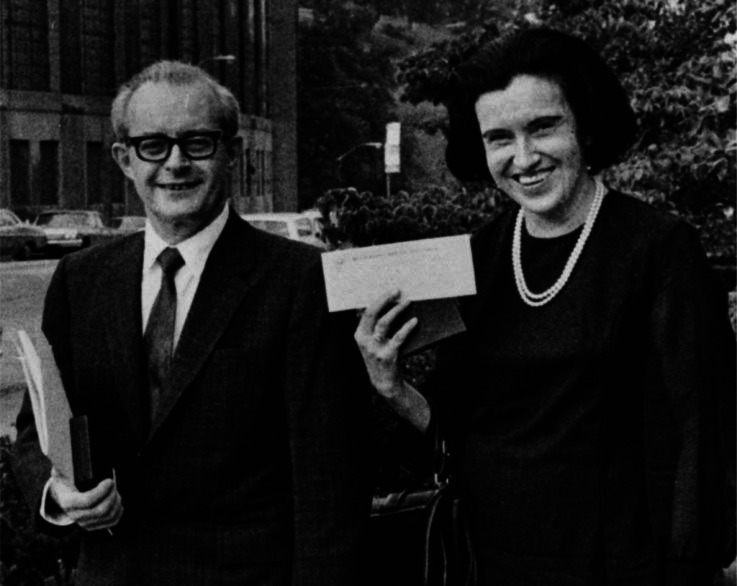
The authors of the principle of the method and the first constructed immunochromatographic rapid test for determination of insulin in human blood plasma (1959) R. Yalou and S. Berson.

The principle of diagnostics based on lateral flow immunoassay (paper chromatography) was first proposed in 1959 by the biophysicist Rosalyn S. Yalow and the physician/endocrinologist Solomon A. Berson (Figure 1).

The first designed system using paraffin paper was a rapid test to determine insulin in human blood plasma [9]. The new principle, soon named LFIA, became a breakthrough technology not only in diagnosis of diabetes mellitus. The formats of new tests progressed rapidly, the paper was replaced by nitrocellulose, and soon the range of clinical laboratory diagnostics was extended by numerous test systems for determining other minor blood analytes (hormones, enzymes, vitamins, and markers of infectious process). As the technology developed, the range of its applications expanded to diagnostics of infectious diseases, cardiovascular diseases [10],[11], cancer biomarkers [12], food pathogens [13], and veterinary diagnostics [14].

Over the following 60 years, several variants of LFIA design were proposed that simplified the method and simultaneously made the test systems more sensitive and selective, affordable, and easy to handle. This allowed their use not only by laboratory staff, but also by other medical specialists and individually by patients for self-monitoring of their health [7],[15]–[17].

The invention of LFIA platforms was mediated by the development of patient-oriented technologies, the shift in the paradigm of patient care culture, and the increasing need for rapidly obtained laboratory information to make urgent decisions in emergency medicine, as well as by the recently introduced global concept of ‘point-of-care testing’ (testing at the site of care) [18]–[20].

To date, the classical microbiological and immunoserological methods, as well as modern diagnostic platforms such as enzyme immunoassay (EIA) and chemiluminescence assay, polymerase chain reaction (PCR), flow cytometry, and mass spectrometry (MALDI), have been used to accurately identify molecular markers. However, these diagnostic tools requiring expensive equipment, long testing time, and qualified personnel are not always available for small local hospitals, especially in conditions of limited budget and decentralized infrastructure of medical units [3],[11],[20],[21].

## 3. LFIA-platforms: types and formats

The potential of these recently introduced efficient technologies consists in the continuous development and improvement of the existing numerous LFIA-platforms, as well as in the creation of multiplex formats and complication of diagnostic goals (such as, e.g., cancer screening). Moreover, the absence of need for special temperature storage conditions contributes to the expansion of the range of their use in developing countries and in sparsely populated and remote regions [15],[16],[21],[22].

Over the decades of application, these test systems have passed the test of time and confirmed their wide availability, high speed of detection, ease of operation and readout of results, and efficient and reliable diagnosis of diseases [21],[23]–[25]. The cost efficiency and easy-to-handle property of these portable diagnostic systems are fully consistent with the world's modern concept of ‘point-of-care testing’ (laboratory testing at the site of treatment). For the above reasons, LFIA have not lost their value today [23]–[25].

Depending on the recognition elements used, LFIA platforms are divided into different types and design formats. Among the formats of this diagnostic strategy are qualitative, semi-quantitative, and quantitative test systems for identifying specific antigens [26],[27], antibodies [28],[29], and fragments of nucleic acids (amplicons) which can be formed during a polymerase chain reaction [12],[30].

**Figure 2.**
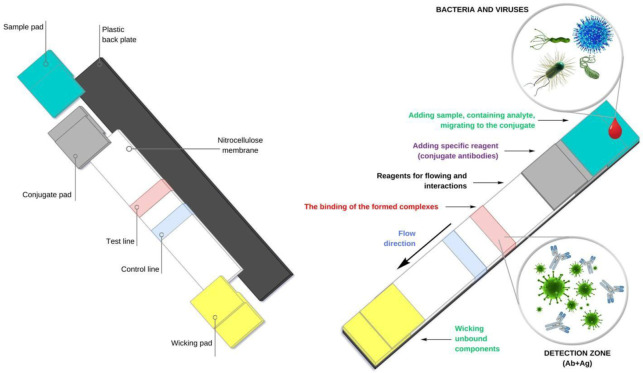
Schematic representation of the lateral flow immunoassay (LFIA) mechanism. A sample containing the test antigen (analyte) is applied to the pad to apply the sample and migrates to the conjugate. A specific reagent with the target analyte migrates to the test line, where it forms a complex with antibodies (author's figure).

The principle of LFIA is simple. A typical test system consists of a plastic base (substrate) coated with overlapping layers of porous membranes containing recognition molecules to interact with the target molecule (Figure 2).

Porous membranes are one of the most important elements of a test system, predominantly made of nitrocellulose. The key parameters that characterize properties of this material are the capillary forces and the ease of binding and subsequent immobilization of proteins involved in further reactions. The pore size of the membranes is from 0.05 to 12 µm, which provides the required rate, time, and uniformity of capillary flow, the most important characteristics determining the quality of test systems [3],[31]–[34].

A liquid sample (biosubstrates) to be analyzed is placed on a sample pad impregnated with a buffer solution, proteins, and surfactants (Figure 2). This part of the test system performs several important functions: even distribution of sample and direction of its movement to the conjugate at a certain rate. Furthermore, the pad acts as a filter to remove unwanted elements of biosubstrates such as red blood cells [10],[35]–[37].

Then the sample is moved by capillary forces along the strip to the pad to release the conjugate containing specific antibodies. The quality of specific antibodies used in the systems and their purification is the most important condition for optimal performance of tests. In LFIA, monoclonal antibodies derived from a hybrid mouse cell line, bound to stained or fluorescent particles (labels), are typically used [2],[32],[38]–[40].

The main requirements for the materials used in LFIA test systems as labels (the list of which is quite wide: they are colloidal gold nanoparticles, colored latex, magnetic and carbon nanoparticles, quantum dots, phosphors, fluorophores, enzymes, etc.) are high stability and low cost.

In addition, the tagging material must be found in very low concentrations and must retain its properties when bound to biorecognition molecules.

The materials listed above fully comply with these requirements; in addition, latex can be made in various colors and used in this form in multiplex systems [3],[13],[41]–[43].

Then the conjugated antibodies bind to the target analyte and migrate to the recognition zone. This part of the strip contains biological components that react with the formed analyte–antibody complex, which is manifested as a colored line in the test zone, while the line in the control zone indicates the correct flow of the substrate [32],[34],[36]. The color intensity of the test line, which is proportional to the analyte content in the sample, is evaluated visually or using special equipment (reader) [44].

To maintain the capillary effect, a cellulose absorbent pad is placed at the distal end of the test strip, which is necessary to remove excess reagents and prevent the reverse flow of the liquid. The application of this pad allows use of a large sample size, thus, increasing the sensitivity of the test [35],[38],[40].

Among other factors that affect test's sensitivity and specificity are chemicals present in biosubstrates that can bind to the system's components and mediate false positives. Sensitivity of a test system is limited by the constant of dissociation of the antibody–antigen conjugate and by colorimetric detection [36],[39]. Therefore, the modern strategies implemented by manufacturers to overcome these limitations are aimed at improving the characteristics of labels used (fluorescent, paramagnetic), which cannot be detected visually but require special devices, i.e. readers, for quantitative analysis [21],[29],[36]. Furthermore, automated detection reduces time expenditures and improves interpretation of results [18],[40].

The modern multiplex LFIA formats are capable of detecting several analytes at a time in a single sample (for example, test systems for detecting narcotic drugs in urine). In this case, the system includes one pad for applying a sample, several (according to the number of tested substances) pads to release conjugate, containing specific antibodies to each of the target analytes, and the same number of test zones to readout the result [21],[26],[29],[43].

Currently, these easy-to-use rapid tests are widely applied to confirm presence or absence of target analytes (e.g., antigens, antibodies, biochemical markers, or amplicons) in biological substrates (urine, serum or plasma, or whole blood) not only in human medicine, but also in environmental research, agriculture, and veterinary medicine [2]–[4],[12]–[14],[18]–[22]. In these fields, the above tests are used to verify pathogens, specific proteins, enzymes, detect chemicals, narcotic drugs, toxins, pollutants, and other substances [10],[14],[23]–[26].

Studies based on LFIA tests are performed at clinical laboratories, sites of medical care, or at home by medical staff or patients themselves. Use of a visual label in the form of gold or carbon nanoparticles or colored latex allows visual qualitative or quantitative (with special equipment available) testing within a few minutes [28],[32],[44],[45].

The assay is based on the antigen–antibody reaction, with biorecognition molecules such as aptamers (the artificial nucleic acids), nucleic acids, proteins or antiligands (specifically aimed at binding target analytes), molecular beacons (special DNA hairpin structure with fluorophore at one end and quencher at the other end), used as antibody, which allows detection of even minor concentrations in the presence of structurally bound molecules [33],[45],[46].

The technological flexibility of the LFIA formats provided creation of many types of test systems based on two key approaches that have gained the greatest popularity among specialists over the past decades:

-noncompetitive (direct) assay (sandwich format) is used to identify analytes having a high molecular weight (HMW) with several antigenic determinants (e.g., р24 antigen, a protein of the HIV nucleotide wall). In this design, result is positive if a color line, absent in case of negative result, appears in the test zone [19],[26],[29]. The most widely known examples of the sandwich format are pregnancy test systems that detect an elevated level of chorionic gonadotropin (from 10 to 25 mIU/mL, depending on sensitivity of tests) in woman's urine at an early pregnancy stage [47];

-competitive assay (inhibition format) is designed for analytes having a low molecular weight (LMW) with a single antigenic site. In this test format, the target analyte blocks the binding sites of antibodies located on the test line, preventing their interaction with the conjugate. In this design, therefore, conclusion is positive in the absence of color line and negative when a color line of any intensity appears in the test zone [48]. The typical examples of this format are drug and toxin test systems.

Each of these formats has advantages and disadvantages depending on analytes being tested, ranges of their critical concentrations, and design of the test system used. The sandwich format usually shows a higher analytical sensitivity (picograms of analyte per 1 mL) compared to competitive test systems (nanograms per 1 mL) [26].

However, at a high concentration of analyzed substance, sandwich systems may show a false negative result associated with the ‘high-dose effect’. The test systems based on the competitive format lack this drawback [47],[48].

Since the 1960s, LFIA test systems have held a firm place in the range of medical diagnostic methods as the most rapid, cost-effective, and simplest tools. Their efficiency has increased significantly with the development of readout technologies and the invention of devices providing transduction of color intensity of indicator lines into a semi-quantitative and quantitative result. In the former case, the result is shown as low, medium, or high; in the latter case, numerical values of analyte concentration depending on the density of test line are displayed [32],[35]–[39].

An example of the quantitative LFIA format is the Seralite-FLC test system for detecting free kappa-(κ-) and lambda- (λ-) chains in blood serum, which displays a numerical value of analyte content in mg/L, as well as the κ/λ ratio, within 10 min [37],[49]. This test system is designed on the basis of highly specific monoclonal antibodies against κ and λ, showing no cross-reactivity with other specific blood proteins [49].

## 4. LFIA tests for diagnosing bacterial and viral infections

Detection and control of infectious diseases is a serious healthcare issue. LFIA testing can be successfully used in diagnostics of infectious diseases [6],[16]–[18],[22],[32].

One of the key trends of development of the versatile LFIA technology is the designing of test systems in a multiplex (multi-purpose) format, which allows detection of several bacterial or viral targets at a time in a single test. This technology is a novel diagnostic approach providing wide opportunities for verification of causative agents [6],[8],[17],[32],[50] (Table 1).

**Table 1.** Examples of modern multiplex formats of LFIA test systems for diagnosing infectious diseases.

Infectious agent	Detectable marker of infectious contamination (target analyte)	Format of test systems LFIA	Refs
*Dengue virus*	Dengue non-structural protein 1 (NS1)	Magneto-enzyme	[51],[52]
*Zika virus*	Zika virus nonstructural protein 1 (NS1)	Smartphone-based fluorescent	[53]
*Chikungunya virus*	SD Bioline, IgM OnSite, IgM	Chromatographic Chromatographic	[54]
*Yellow fever (YF) virus*	YF non-structural protein 1 (NS1)	Chromatographic	[55]
*Ebola virus*	Antigen Ebola virus ReEBOV VP40	Magneto-enzyme	[56]
*Dengue virus*	Ig G/ IgM	Multiplex	[42]
Yellow fever (YF) virus	Ig G/ IgM	Multiplex	[42]
Ebola virus	Ig G/ IgM	Multiplex	[42]
Human immunodeficiency virus (HIV)	Anti-HIV IgG	Multiplex	[41]
Hepatitis C virus (HCV)	Anti-HCV IgG	Multiplex	[41]
Hepatitis B virus (HBV)	Hepatitis B e-antigen (HBeAg)	Monoplex	[57]
Human Immunodeficiency Virus (HIV-1)	HIV-1 p24 antigen	Monoplex	[58],[59]
Foot-and-mouth disease virus	Antigen detection for all 7 serotypes for types O, A, C and Asia1	Multiplex	[60]
Respiratory viruses	Human adenovirus, influenza A H1N1 virus	Magnetic SERS-based LFIA (FeO @ Ag)	[61]
Newcastle disease virus (NDV)	Amplification (RPA)-nucleic acid lateral flow (NALF) immunoassay	Multiplex RPA-NALF	[62]
Infectious bronchitis virus (IBV)	Amplification (RPA)-nucleic acid lateral flow (NALF) immunoassay	Multiplex RPA-NALF	[62]
Human Polyomavirus BK (BKV)	DNA BKV	Monoplex sandwich-type	[63]
*Bordetella pertussis*	Anti-toxin pertussis IgG	Fluorescent Eu-nanoparticle reporters	[64]
*Staphylococcus aureus*	Staphylococcal enterotoxin B	SERS-based lateral flow immunoassay	[65]
*Streptococcus pyogenes*, Group A	*S. pyogenes (A)*	SERS-based lateral flow immunoassay	[66]
*Yersinia pestis*	F1 capsular antigen	Monoplex	[67]
*Escherichia coli* O157: H7	*E. coli* O157: H7	Sandwich models	[68]
*Listeria monocytogenes*	*L. monocytogenes*	SERS-based lateral flow immunoassay	[69]
*Salmonella typhimurium*	*S. typhimurium*	SERS-based lateral flow immunoassay	[69]
*Helicobacter pylori* (HpSA-test)	Antigen *H. pylori* HpSA	Monoplex	[70]

The first commercial combined LFIA test system was successfully demonstrated by C.S. Jørgensen with co-authors in 2015 [25]. It allowed efficient detection of *Streptococcus pneumoniae* and *Legionella pneumophila* antigens in the urine. This even more increased the popularity of the versatile LFIA technology, which, in the sandwich assay format, is equally efficient for both HMW antigens of microorganisms and antibodies to them in biosubstrates and for LMW analytes [8],[16],[23],[24].

## 5. Advantages and disadvantages of LFIA test systems

Today, test systems based on the immunoassay technology, in both the standard and multiplex formats, represent a major segment of the market of laboratory-based rapid diagnostics [18],[23],[24],[32]. Many of them are successfully used within the framework of the ‘point-of-care testing’ (POC) program [3],[8],[11],[15],[71].

Result of immunochromatographic tests can be verified with the naked eye. In addition, these tests have advantage such as low cost of operation, easy handling, and operation without additional equipment [23],[27],[28],[32].

When molecular markers of an infectious process are detected using LFIA, this usually requires confirmation by an independent method. Therefore, immunoassay technologies are suitable mostly for primary screening [6],[8],[17],[72]. In addition, despite the obvious attractiveness of the LFIA formats, their limitations a great while held back the expansion of the practical use of these diagnostic platforms for clinical laboratory diagnostics (Table 2).

**Table 2.** Limitations and advantage of LFIA test systems.

Advantage	Refs	Limitations	Refs
Cheap, rapid, affordable, and easy-to-handle tests. Small size of analyzed sample	[3],[27],[40]	Tests are suitable for only primary screening and require confirmation of positive results by independent methods	[27],[29],[40],[62]
Single-stage analysis with no need of additional reagents. Long shelf-life of test systems. Do not need qualified specialists.	[23],[32],[40]	Special equipment (scanners, reflectometers, CCD cameras) and software are required to obtain quantitative results.	[23],[28],[32],[47]
Test systems can be used in quantitative and semi-quantitative formats. Allow detection of proteins, haptens, nucleic acids, or amplicons.	[12],[31],[37]	Technological improvements of the method increase the cost and duration of analysis. In the competitive format, response negatively correlates with concentration.	[10],[29],[37],[42]
Do not require special temperature conditions for storage. Do not require additional special equipment when a high-quality format is used.	[3],[18],[28],[32]	Possible technical errors during application of specimen may affect the accuracy and reproducibility of result. Pores may be blocked by components of the sample tested.	[3],[13],[32],[54]
Do not need qualified specialists. Can be used by general physicians or patients at home. Visual result is clear and easily readable. Opportunity of multiplexing.	[4],[10],[13]	Increase in sensitivity of tests is based on the use of gold, silver, or enzyme nanoparticles, which limits shelf-life, increases the cost of analysis, and breaks the principle of one-stage use.	[4],[28],[51],[55]
Tests are usually distributed as kits with a set of all items needed to perform them.	[13],[24],[26]	Tested specimen must be in the form of solution. Preliminary dissolution of dry samples is mandatory.	[13],[33],[52],[59]
Sensitivity of test systems can be increased by using plasmon resonance, surface-enhanced Raman scattering (SERS), chemiluminescent or fluorescent labels.	[12],[25],[33],[35]	If the analyte content in the solution is low, the specimen needs to be concentrated. Inaccurate selection of sample size reduces the accuracy of result.	[12],[23],[42],[58]
It is possible to detect antibodies (IgM and IgG) and pathogen antigens.	[23],[28],[37],[38]	Analysis time depends on viscosity of the sample tested.	[23],[28],[47],[62]

However, modern platforms of immunochromatographic systems, devoid of many drawbacks, have successfully proven themselves not only in medicine, but also in veterinary and environmental research. In addition, the high sensitivity, safety, and ease of use of LFIA test kits are critical in the elimination of epidemic outbreaks of dangerous infections.

R. Nouvellet et al. [73] give an example of the successful application of chromatographic analysis in the elimination of the Ebola epidemic in 2014–2015 in West Africa. At the initial stage of the epidemic, according to the WHO recommendation, the diagnosis of Ebola was based solely on the results of reverse transcriptional polymerase chain reaction (RT-PCR), which detected viral RNA in serum or plasma. However, these diagnostics was slow and expensive (2–6 hours at a cost of US 100$), and blood storage required maintaining a cold chain, which was problematic in Africa. The extreme nature of the epidemic has prompted WHO to call for fast and inexpensive test systems that do not require special instruments and equipment. Such test systems are chromatographic strips for the detection of the Ebola antigen ReEBOV VP40. Ultimately, it was recognized that rapid and accurate diagnostics was critical to successfully containing and eliminating the Ebola outbreak [73].

Further research is currently conducted to address some of the major drawbacks of LFIA test systems, especially as regards obtaining quantitative results and documenting them. They can be digitized using scanners or cameras with special software that would allow also recording the result and transmitting it at a distance. However, technological improvements will require more sophisticated hardware and will eventually increase the cost and duration of analysis.

## 6. Modern technologies for the quantitative interpretation of the results of LFIA analysis

Analyzing the advantages and limitations of LFIA technology, many authors for many years have pointed out a significant drawback (‘key failure’) of these diagnostic tools–visual assessment and qualitative conclusion (yes/no), which limits the objectivity and informational value of these analyzes [6],[74].

Advanced strategies of the development of LFIA testing technologies are capable of providing reliable quantitative information about the content of the target analyte in biosubstrates. In 2019, a fairly detailed reviews on this topic was published [6],[74],[75], so the author will limit himself to only a conceptual discussion of these modern approaches.

Traditionally, LFIA tests were considered as diagnostic tools for the qualitative analysis of the presence (or absence) of the desired analyte (marker) or its content exceeding a certain threshold. The result was assessed visually by comparing the staining of the test area with the control area [76].

The need to improve immunochromatographic technologies was associated with an increase in the relevance of a quantitative assessment of the content of markers of the pathological process during dynamic monitoring of the effectiveness of therapy, the patient's condition or the environment. At the same time, the inclusion of instrumental registration in LFIA tests did not make them more complicated.

In recent years, technological designs of various detecting devices have been proposed for quantitative assessment of immunochromatography, based on various principles of detection, ranging from the first reflectometric instruments to modern portable digital cameras [75],[76]. These detectors allow not only to quantitatively analyze biological samples, but also to store and transmit on-line results for a remote objective conclusion [74],[77],[78].

An important advantage of the LFIA technologies that are currently being actively developed are sensor and array-based platforms. The specialized quantitative analysis tools LFIA tests are divided according to the principles of recording the result. Thus, in the last decade, commercial models of open and closed detectors have appeared, based on optical data processing, which allow reading not only color stripes, but also fluorescent labels [6],[76],[79] (Table 3). In addition, the advent of related software products for smartphones allows these individual mobile devices to be used to obtain rapid quantitative results without the use of additional equipment [75],[80]. The use of mobile device platforms in quantitative LFIA formats to accurately record test results increases the efficiency of the clinic, epidemiological surveillance and disease control at the system level, and ultimately reduces the time it takes to initiate specialized treatment. In cases of suspected bacterial or viral infections, the use of mobile devices in LFIA testing allows for the rapid detection of isolated cases and a timely public health response.

For example, A. Nsabimana and colleagues in a recent study assessed the feasibility and effectiveness of this smart technology LFIA platforms for recording quantitative HIV test results in 2,190 patients in urban and rural Rwanda (East Africa) in 3 hospitals.

In recent years, with the emergence advent of related software products for smartphones allows these personal mobile devices to be used to obtain rapid quantitative results without the use of additional equipment [75],[80]. The use of mobile device in quantitative LFIA formats to accurately record test results increases the efficiency of the clinic, epidemiological surveillance and disease control at the system level, and ultimately reduces the time it takes to initiate specialized treatment. In cases of suspected bacterial or viral infections, the use of mobile devices in LFIA testing allows for the rapid detection of isolated cases and a timely and timely public health response.

For example, A. Nsabimana and colleagues [81] in a recent study assessed the feasibility and effectiveness of this smart technology LFIA platforms for recording quantitative HIV test results in 2,190 patients in urban and rural Rwanda (East Africa) in 3 hospitals.

**Table 3.** Examples of modern LFIA detector platforms for quantifying results (based on [6], with actualization and additions).

Detection platforms principle	Company/Country	Detector Model	Mode of Measurements	Company website
Optical	Axxin/Australia	AXXIN AX-2X	Colorimetry, fluorimetry	axxin.com
	Bio-AMD/United Kingdom	Digital Strip Reader	Colorimetry	bioamd.com
	BioAssay Works/United States	Cube-Reader	Colorimetry	bioassayworks.com
	Hamamatsu/Japan	Immunochromato-Reader C11787	Colorimetry, fluorimetry	hamamatsu.com
Magnetic Labels (Magneto-enzyme)	Magna BioSciences/United States	MICT^®^ Bench-Top System	Open System	magnabiosciences.com
	Magnasense Technologies/Finland	Magnasense's Magnetometric Reader	Open System	magnasense.com
	VWR International/United States	FoodChek™ MICT System	Closed System	vwr.com

As an alternative to mobile devices, it is proposed to use standard office scanners, which are already actively used in the quantitative analysis of the results of electrophoresis of protein fractions of blood [78],[82].

In modern formats of immunochromatographic test systems, magnetic particles are used as labels. Their registration in a magnetic field is not affected by the color of the bioassay or the colored components of the substrates. Magnetic recording detectors for quantitative sidestream analysis are more efficient and sensitive and have proven themselves in the commercial market [51],[61] (Table 4).

**Table 4.** Examples of quantitative lateral flow assays and their analytical parameters.

Detecting Device and Construction	Target Analytes	Range of Concentration Measured	Refs
Dual LFIA with iPhone 5s	*Salmonella enteritidis*	20–10^7^ CFU/mL	[7]
	*E. coli* O157:H7	34–10^7^ CFU/mL	
UC-LFS platform	Brain natriuretic peptide	5–100 pg/mL	[31]
	Suppression of tumorigenicity 2	1–25 ng/mL	
Smartphone's ambient-light-sensor-based reader (SPALS-reader)
	Cadmium ion	0.16–50 ng/mL	[35]
	Clenbuterol	0.046–1 ng/mL	
	Porcine epidemic diarrhea virus	0.055–20 µg/mL	
Electrochemical detection	Human chorionic gonadotrophin (HCG)	25–50 mIU HCG in serum	[82]
Magneto-enzyme	Dengue virus non-structural protein 1 (NS1)	0, 1–0,25 ng/ml	[51]
Magnetic SERS (Raman scattering-based LFIA)	Human adenovirus,	from 50 PFU/mL	[61]
	Influenza A H1N1 virus	from 10 PFU/mL	
Raman scattering-based LFIA (SERS-LFIA)	Streptococcus pyogenes, Group A	0,2–100 KOE/mL	[66]
	Listeria monocytogenes	10^2^–10^7^ KOE/mL	[69]

Electrically conductive metal nanoparticles or oxidizing enzymes can be used as markers in the design of modern test strips. The principle of signal measurement in such systems is based on fluctuations in current, voltage or resistance (amperometry, potentiometry or conductometry) [82].

And, finally, surface enhanced Raman spectroscopy (SERS) is increasingly used in modern models of quantitative detection of LFIA to increase the sensitivity of the immunochromatographic method [61],[65],[69]. These formats allowing the detection of specific biomarkers of infections (antibodies, peptides, or DNA) conjugated with gold nanoparticles [65],[69].

Modern SERS platforms for sandwich immunoassay using paper test strips have proven to be in great demand for the quantitative detection of infectious diseases and, in particular, viral infections, including COVID-19.

## 7. Lateral flow immunoassay and diagnosis of COVID-19

At the end of 2019, a new coronavirus infection COVID-19 appeared in China, the subsequent spread of which around the world assumed the character of a pandemic [83]–[85]. In this regard, the creation of platforms for the effective diagnosis of this disease has become especially relevant for public health [84],[86]–[88].

It is known that COVID-19 belongs to the *Coronaviridae* family of RNA-containing coronaviruses that cause acute respiratory infections in humans of varying severity (from asymptomatic or mild to severe pneumonia) [84],[86],[87]. In this case, the main pathogenetic targets are the respiratory system, the gastrointestinal tract, the liver and the central nervous system, the defeat of which has become a characteristic feature and others coronavirus infections (Middle East Respiratory Syndrome, MERS, and Severe Acute Respiratory Syndrome, SARS) [85],[88],[89].

Before the emergence in the XXI century of epidemic outbreaks of infections (SARS in 2002–2004 and MERS in 2012 caused by β-coronaviruses MERS-CoV [80],[81],[84] and SARS-CoV [86],[87], these pathogens were not considered highly pathogenic to humans. They circulated in the human population, causing only sporadic cases of diseases that occur, as a rule, in a mild form [85],[88]. Epidemic outbreaks of SARS and MERS, and especially COVID-19, have changed the current view of the pathogenicity of coronaviruses and the epidemiology of new infections, as well as the potential perspective of emergence new outbreaks [86],[89]–[91].

The etiological cause of the 2019 pandemic was the new SARS-CoV-2 virus (Severe acute respiratory syndrome coronavirus 2), which in half a year caused hundreds of thousands of deaths and caused disease in millions of the world's inhabitants, caused chaos and a drop in the level of the world economy, and in the world community–fear and anxiety for the future of mankind. According to a WHO report, as of July 1, 2020, the number of COVID-19 cases in the world exceeded 10 million, and the quantity of deaths was over 500 thousand [90].

Like other β-coronaviruses, the SARS-CoV-2 gene contains specific RNA sequences encoding 27 proteins. Among them, 15 non-structural proteins that provide virus replication [87],[92]–[94] and 12 structural proteins are distinguished. For SARS-CoV-2, antibodies have been detected that recognize three of the four SARS-CoV-2 proteins exposed on the surface of the viral capsid: the nucleocapsid (N), envelope (E), and spike (S) proteins [95].

One of the most important directions in the strategy of combating a new infection has become the need for mass laboratory screening of populations at high risk of infection. The need for timely and high-quality laboratory diagnosis of patients infected with SARS-CoV-2 has become the main priority in eliminating the pandemic and introducing quarantine measures [87],[90],[96],[97].

In these conditions, the creation of quick, effective and inexpensive tools for the diagnosis of COVID-19, on the one hand, has become an essential component of the fight against a new infection, and on the other, it has been focused on the experience of eliminating previous coronavirus infections SARS and MERS [86],[88],[91],[92].

However, both in cases of elimination of SARS and MERS infections, and in the diagnosis of COVID-19, public health faces the same task. It is associated with determining the role and place of various diagnostic platforms for screening, diagnosis and monitoring of new coronavirus infections: RT-PCR, RT-LAMP, ELISA and LFIA, taking into account the advantages [87],[89],[91],[98].

The isolation of the pathogen culture in its pure form, which is the gold standard for the diagnosis of viral infections, is a laborious and lengthy process associated with working in a special laboratory that has the appropriate permission to work with biologically dangerous pathogens [94],[98].

Molecular testing of real-time polymerase chain reaction (RT-PCR) coronavirus infections, which is widely used in diagnostic virology, is a highly sensitive and recognized biomedical method [29],[87],[92],[98]. However, the full cycle of the study takes about 2 hours, sample preparation/extraction is required which takes 2–3 hours, and it is necessary special equipment, as well as trained specialists in laboratory diagnostics, which limits its use in COVID-19, despite the fact that PCR diagnostics are used throughout the world during a pandemic to detect SARS-CoV-2 [89],[93],[97].

The main disadvantages of this method in the diagnosis of infections are derived from the principle of the RT-PCR method, which provides for the detection of coronavirus RNA of only a specific type (SARS-CoV-2) and at a certain period of the disease [80],[87],[92]. Therefore, the results may be negative when examining convalescing patients cleared of the pathogen, as well as in patients with other infections [88],[98]. In addition, a false-negative result may be due to the uneven distribution of the virus in the respiratory system (sputum, nasopharyngeal secretion, pharynx), as well as (which seems inevitable in conditions of a huge flow of biological samples) ignoring the standards for sample collection [29],[86],[93],[98].

Recently, for screening and monitoring studies in coronavirus infections, the faster (30 mins–1 h) and economical molecular method of isothermal amplification of nucleic acids (LAMP and RT-LAMP) has become increasingly popular [34],[89],[99]–[101]. This diagnostic technology involves the use of simple-to-operate equipment with the ability to visually evaluate the result [69],[82]. This diagnostic method was proposed in 2000 and managed to prove itself in epidemics of SARS and MERS infections [69],[79],[80], as well as during the COVID-19 pandemic as a screening tool for remote regions and rural hospitals [86],[88].

The disadvantages of LAMP testing are the researchers' lack of sufficient experience in using the method in the conditions of epidemic outbreaks and emergency situations associated with SARS, MERS and the COVID-19 pandemic, as well as of the clinical interpretation of the results [34],[89],[95]. Like RT-PCR, RT-LAMP only detects the presence of viral genetic material and does not indicate the facts of previous infection and subsequent recovery of the patient [85],[86].

On the contrary, serological tests in the most common formats of enzyme-linked immunosorbent assay (ELISA) and of rapid tests LFIA, are widely known and have proven themselves as simple and cost-effective diagnostic platforms for coronavirus infections [83]–[89]. In contrast to molecular genetic diagnostic platforms, serological tests can detect not only viral contagion of patients, but also evaluate the response of the immune system and provide the necessary information for making organizational decisions [89],[91],[92],[96],[97].

Based on immuno-serological testing, these diagnostic technologies can provide faster and more detailed epidemiological information. For example, a full cycle of an ELISA study takes at least several hours to complete and requires special equipment in a clinical laboratory [83]–[86], while analysis using LFIA test systems requires 10 to 20 minutes [93]–[96],[98]–[100].

In the context of the diagnosis of SARS, MERS, and COVID-19 infections, LFIA rapid tests were most often used to test for the presence of patient antibodies (IgG and IgM) or viral antigens [93],[95],[98],[100]. Thus, using these tests, it is possible to identify not only infected patients, but also conduct retrospective diagnostics for those patients who have suffered asymptomatic disease, have recovered and currently have a certain degree of immune defense [97],[99],[100].

For example, J. Wu et al. [88] performed a retrospective study of the dynamics of the appearance of antibodies to SARS-CoV-2, the time-dependent sensitivity of four LFIA test systems in patients, to diagnose the significance of rapid serological tests in the management of patients with COVID-19, the diagnosis of which was established by molecular testing (RT-PCR). It turned out that 3 weeks after the onset of symptoms of the disease, all tests revealed antibodies (IgM and IgG), and the sensitivity and specificity was 100%. Moreover, in patients with COVID-19 complicated by pneumonia, an earlier appearance of antibodies against SARS-CoV-2 was detected [88].

In another study, Z. Chen and colleagues [92] report the development and testing of a new LFIA system for the detection of anti-SARV-CoV-2 IgG antibodies in human serum. The design features of the new test system are associated with the use of lanthanide-doped polystyrene nanoparticles and the recombinant nucleocapsid phosphoprotein SARS-CoV-2, placed on a nitrocellulose membrane to capture specific antibodies. The whole analysis process takes 10 minutes [92].

In accordance with the LFIA technology, infectious markers can be detected in the blood (antibodies), saliva, and the upper respiratory tract secretion throughout the infection cycle (contamination, incubation period, high season, recovery) [94],[101]–[103]. Unlike molecular methods, the use of LFIA in-house rapid tests does not require special training of specialists and can be used for screening studies in the field, at railway stations, airports, at unequipped diagnostic points, and in rural hospitals [95],[97],[104]–[106].

Despite the fact that the use of individual LFIA test systems turned out to be more expensive compared to ELISA in practical applications for the diagnosis of SARS, MERS and COVID-19 infections, their use is justified by the clinical advantages of this diagnostic platform [97],[100],[107].

During the fight against the COVID-19 pandemic, many different diagnostic systems were developed and proposed based on the principles of side-stream immunoassay, which differ in sensitivity and specificity [98],[108]–[111]. Some of the proposed platforms passed the necessary expertise and received permission for use in clinical practice (Table 5). Reliable efficacy and high sensitivity in detecting SARS-CoV-2 specific antibodies using immunochromatographic test systems shows that LFIA technologies can be a useful diagnostic tool in addition to molecular methods for diagnosing COVID-19. The international experience of using serological tests based on lateral flow immunoassay to eliminate epidemic outbreaks of coronavirus infections has shown the importance and necessity of this diagnostic tool. The most rational use of LFIA is mass screening of the population from risk groups, as well as patients with asymptomatic form of the disease. All positive results must be verified by quantitative molecular genetic methods.

**Table 5.** LFIA test-systems approved for diagnostics COVID-19 [112].

Country-developers and producer companies	Sensitivity/ specificity of the test-systems (%)	Description of the test system	Biosubstrates used for diagnosis, analysis time	Links
US / China, *Cellex Inc.*	93,8/95,6	IgM/IgG is detected by SARS-CoV-2 protein nucelocapside	Serum, plasma or whole blood (K2-EDTA, sodium citrate), 20 min	[113]
US, *ChemBio*	92,7 (IgM) и 95,9 (IgG)/99,0 (IgM и IgG)	IgM/IgG is detected by SARS-CoV-2 protein nucelocapside	Finger or vein whole blood, serum and plasma (lithium heparin, K2-EDTA), 15 min	[114]
*US, Autobio Diagnostics Co. Ltd. (+ Hardy Diagnostics)*	95,7 (IgM) и 99,0 (IgG)/99,0 (IgM и IgG)	IgM/IgG is detected by SARS-CoV-2 antigens	Finger or vein whole blood, serum and plasma (heparin, K2-EDTA), 15 min	[115]
US / China, *Healgen Scientific LLC*	96,7 (IgG), 86,7 (IgM), 96,7 comb./98,0 (IgG), 99,0 (IgM), 97,0 comb.	IgM/IgG is detected by SARS-CoV-2 antigens	Finger or vein whole blood, serum and plasma (heparin, K2-EDTA), 10 min	[116]
China, *Hangzhou Biotest Biotech Co., Ltd*	92.5 (IgM), 91.56 (IgG)/98.1 (IgM), 99.52 (IgG)	IgM/IgG is detected by SARS-CoV-2 recombinant spike protein receptor binding domain	Finger or vein whole blood, serum and plasma (heparin, K2-EDTA), up to 20 min	[117]
China, *Biohit Healthcare (Heifei) Co. Ltd.*	33.0 (IgM, days 1–7), 56.6 (IgG days 8–14), 83.0 (IgM days 8–14), 96.2 (IgG days 15+), 97.7 (IgM days 15 +)/99.5 (IgM), 100.0 (IgG)	IgM/IgG is detected antibodies by SARS-CoV-2 recombinant N-protein antigen and mouse anti human IgM/IgG antibody	Samples for human serum, plasma or whole blood (heparin, K2-EDTA, and sodium citrate), 15 min	[118]
China, *Hangzhou Laihe Biotech Co., Ltd*	100,0 (IgM, 0–6 days), 85.7 (IgM, 7–14 days), 76,0 (IgG, 7–14 days), 99.25 (IgM, 14+ days), 98.5 (IgG, 14+ days) / 99.43	IgM/IgG is detected by SARS-CoV-2 antibodies. The target antigen is the S1 region of the spike protein.	Samples for human serum, plasma or whole blood (heparin, K2-EDTA, and sodium citrate), 15 min	[119]
US / China, *Aytu Biosciences / Orient Gene Biotech*	87.9 (IgM) & 97.2 (IgG)/100,0 for IgG and IgM	IgM/IgG is detected antibodies by SARS-CoV-2 antigen.	Samples for human serum, plasma or whole blood, 10 min	[120]

Note: * - according to the Center for Health Security at J. Hopkins University [112].

## 8. Modern technology development strategies of LFIA diagnostics

Some of the above-discussed innovations are associated with variations in the nature of labels used, as well as with technical improvements in the quantitative format of conclusion made. Some of the new strategies are based on a combination of colloidal gold nanoparticles with enzyme (such as horseradish peroxidase), which causes a catalytic amplification of signal [50],[51]. Other methods of signal amplification (1000-fold or more), as well as increase in sensitivity of test systems, are associated with the use of laser detection (plasmon resonance, surface-enhanced Raman scattering), chemiluminescent or fluorescent labels [12],[30],[45],[65].

A noteworthy format of LFIA to detect increase in myoglobin concentration, based on a sandwich system, was proposed by К. Edwards with co-authors [43]. In the proposed system, immobilized antibodies conjugate with streptavidin and are detected with a specific fluorescent dye (sulforodamine B) encapsulated in liposomes, which facilitates signal generation [43].

A number of promising innovations for multiplexing of the LFIA technology have been successfully tested in recent years [21],[44],[47]. Thus, there are test systems that include colloidal gold nanoparticles and oligonucleotides for the simultaneous detection of antigens and antibodies [21],[44] and the use of two conjugate pads for the simultaneous detection of two proteins [44]. Furthermore, a combination of LFIA with electronic computing units provides a response in the format of ‘OR’ and ‘AND’ logic gates [47].

As biomedical applications expanded, the requirements for LFIA systems grew steadily. They primarily concerned the improvement of sensitivity, reproducibility and the possibility of multiplexing, as well as increasing the objectivity of the quantitative assessment of results, which has recently been associated with laboratory information systems.

Modern technological trends in the improvement of immunochromatographic test systems are associated with obtaining high-sensitivity results with low constant of variance (CV). Improvements in sensitivity would allow assay LFIA systems to be applied in areas where larger clinical immunoassay systems, and methodologies such as PCR, are considered the gold standards. It is known that PCR diagnostics is of key importance for the diagnosis of bacterial and viral infections, biomedical research, food safety assessment, and environmental monitoring. At the same time, there was an urgent need for a simple, fast and cost-effective method without the use of complex and expensive equipment and reagents that are not quite available in conventional laboratories to detect fragments of nucleic acids (NA). This has led to the creation of paper-based analytical platforms that combine highly sensitive molecular genetic technologies with the speed and convenience of lateral flow technologies.

Over the past 10–15 years, a significant number of platforms have been proposed that have been developed for the detection of HA fragments using lateral flow technology and PCR [121]–[125]. These methods have simplified PCR technology, eliminating electrophoresis for confirming the presence of nucleic acid after DNA amplification, and of purchase of expensive equipment for molecular genetic analysis [126]–[128].

The very idea of detecting DNA fragments using LFIA analytical tools is not new and was successfully implemented in practice at the end of the 20th century [129]. Today, this technology is actively used in many areas of biomedicine, such as veterinary diagnostics, food and environmental monitoring, and diagnostics of plant diseases [122],[124],[125].

In new technologies that combine the capabilities of PCR and the advantages of LFIA, an amplified double-stranded sequence that is specific to the target microorganism is captured on paper immunochromatographic strips in the antibody-antigen format. In this pair, the antibody is specific to the label (for example, to biotin or streptavidin) and the Antigen is a labeled amplicon. The use of nitrocellulose strip as an immunosorbent and analytical platform allows one-step, fast and inexpensive analyzes (123,126,127). NA detection is performed using primers with two different labels (for example, streptavidin + or biotin +), and reporters (for example, avidin-labeled gold nanoparticles) provide visualization with the naked eye.

The developed new formats that constructively combine PCR and LFIA technologies are presented in two types: Nucleic Acid Lateral Flow Immunoassay (NALFIA) and Nucleic Acid Lateral Flow (NALF). The fundamental difference between these formats lies in the method of NA determination: direct, using reporter oligonucleotide probes (NALF) or previously labeled NA with hapten labels (digoxigenin, fluorescein, biotin, using reporter antibodies or streptavidin. Accordingly, the new analytical platforms were called PCR-NALF and PCR-NALFIA [122],[124],[126],[127].

Thus, M. Jauset-Rubio with colleagues [130] are report on the development of a point-of-care PCR-NALF test for the direct detection of isothermally amplified DNA. The detection limit (1 × 10^−11^ M or 190 amol), which is equivalent to 8.67 × 10^5^ DNA copies, while the entire study cycle (amplification and detection) lasted less than 15 minutes at 37 °C.

In another study, S. Pecchia & D. Da Lio [126] proposed a test system in the PCR-NALFIA format for the detection of *Macrophomina phaseolina* in different types of infected soils and for pathogen detection and identification in plant tissues.

Thus, the evolution of LFIA technology occurs both in the direction of improving the analytical performance of these diagnostic tools and the emergence of new platforms that combine the advantages of various methods [124],[126],[130]. The possibilities of quantitative detection, analysis multiplexing, as well as the emergence of modern LFIA formats combined with PCR, create unprecedented laboratory diagnostic opportunities for the development of the POC concept. The future perspective is possibly related to the opportunity of a complete replacement of the PCR method, which requires trained specialists and special equipment, with LFIA platforms with the function of recombinase polymerase amplification.

## 9. Conclusion

Over the 60-year history of application of immunoassay, diagnostic technologies have become an indispensable tool in medicine, veterinary, and ecology, firmly occupying the position of the most demanded and popular rapid tests that fully conform to the modern global concept of ‘point-of-care testing’ [15],[16],[27].

The principle of the lateral flow immunoassay method by R. Yalow and S. Berson has remained unchanged for the past decades, despite the numerous latest LFIA formats proposed to improve its sensitivity and specificity. Immunoassay now becomes increasingly widespread in the world's healthcare systems. Today, this does not necessarily mean substitution of centralized laboratories by ‘point-of-care testing’ technologies, as LFIA platforms occupy only certain position in diagnostics of emergency conditions and monitoring of patients' health.

The main advantages of the method–simplicity and availability combined with its high efficiency–have always been decisive when choosing between tools for diagnostic screening in conditions of limited budget and low access to well-equipped laboratories or medical units. However, the simplicity of LFIA platforms contradicts the complex goal of optimizing the method to make it more sensitive, multiplexed, and quantitative. Therefore, the modern strategies implemented by designers of the method focus on empirical selection of materials for membranes, purity of reagents (antibodies, buffer systems, blocking reagents), and design of test systems [50].

The latest innovations aimed at improving the analytical characteristics of the LFIA technology are interesting, promising, and can provide these platforms with additional advantages (e.g., integration into the ‘chip-based laboratory’ design) [66]. Nevertheless, most of them increase cost of test systems, their complexity, and, thus, reduce availability of these remarkable technologies, a property that has made them popular and attractive for the recent six decades.
